# Cytokine adsorption in patients with severe COVID-19 pneumonia requiring extracorporeal membrane oxygenation

**DOI:** 10.1186/s13054-020-03130-y

**Published:** 2020-07-14

**Authors:** Marina Rieder, Tobias Wengenmayer, Dawid Staudacher, Daniel Duerschmied, Alexander Supady

**Affiliations:** 1 Department of Medicine III (Interdisciplinary Medical Intensive Care), Medical Center, Faculty of Medicine, University of Freiburg, Freiburg, Germany; 2 Department of Cardiology and Angiology I, Heart Center, University of Freiburg, Hugstetter Strasse 55, 79106 Freiburg, Germany

The treatment of patients with severe COVID-19 requiring support with veno-venous extracorporeal membrane oxygenation (vv-ECMO) is particularly challenging from a medical point of view and consumes a tremendous amount of human, physical, and financial resources. Recommendations for initiation of vv-ECMO in COVID-19 are being developed, though under continuous review [1, 2]. However, despite all therapeutic efforts, these critically ill patients have a high mortality rate according to studies published so far [3, 4].

At our hospital, a major referral center for extracorporeal support, we have treated several COVID-19 patients with vv-ECMO. Knowing interleukin-6 (IL-6) as a predictor of negative outcome, some patients received cytokine adsorption using the CytoSorb® adsorber (CytoSorbents Europe, Berlin, Germany) shortly after initiation of ECMO for up to 72 h [5]. Based on experience in septic patients, the adsorber was exchanged every 24 h [6]. Integration of the adsorber in the ECMO circuit was feasible and safe. Preliminary data from eight cases (4 patients receiving ECMO with cytokine adsorption, the remaining 4 received ECMO without cytokine adsorption) shows that cytokine adsorption may result in a more pronounced decrease of IL-6 after initiation of ECMO (Fig. 1).

**Fig. 1:**
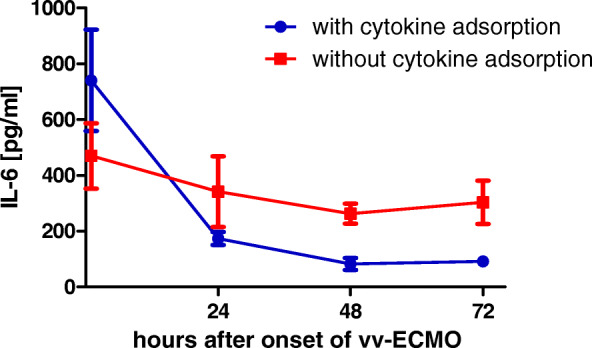
IL-6 levels within the first 72 h after onset of vv-ECMO in 8 patients. Four patients received vv-ECMO without cytokine adsorption (red), and 4 patients vv-ECMO with cytokine adsorption (blue). Data are presented as mean ± SEM

Whether this observation can be confirmed in larger cohorts and the degree to which this effect is caused by the adsorber instead of just being a sign of general clinical improvement warrants further investigation. Finally, whether this translates into improved clinical outcome remains to be shown, either.

Therefore, we set up a clinical trial to further examine this preliminary observation. The CYCOV-II study (cytokine adsorption in patients with severe COVID-19 pneumonia requiring extracorporeal membrane oxygenation) is a randomized, controlled, open-label intervention, multicenter trial comparing cytokine adsorption in ECMO treatment for COVID-19 with a control group receiving standard ECMO treatment without cytokine adsorption (Fig. 2; NCT04385771).

**Fig. 2:**
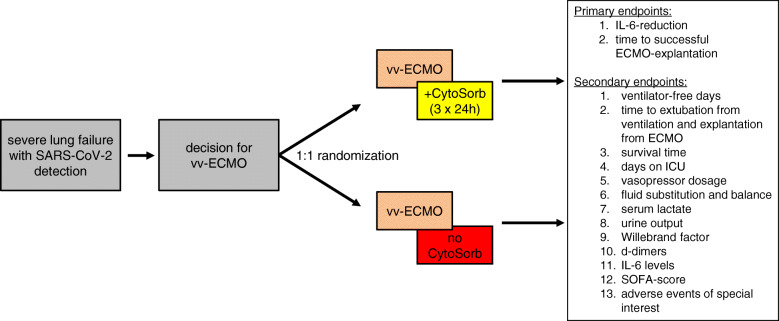
Graphic representation of the study design for the CYCOV-II trial

In all participating centers, we plan to randomize all COVID-19 patients receiving vv-ECMO into the study groups. Cytokine adsorption will be performed for 72 h. The primary endpoints of the study are (1) IL-6 reduction by 75% or more after 72 h as compared to the baseline measurement and (2) time to successful ECMO explantation within 30 days after randomization. Further clinically relevant endpoints, such as ventilator free days, days on intensive care unit, and overall survival time, will be assessed as secondary endpoints.

With this study, we expect to clarify whether cytokine adsorption is beneficial in severely affected COVID-19 patients requiring vv-ECMO support.

## Data Availability

All data will be available from the corresponding author on reasonable request.
